# Heterologous expression and characterization of an Arabidopsis β-l-arabinopyranosidase and α-d-galactosidases acting on β-l-arabinopyranosyl residues

**DOI:** 10.1093/jxb/erx279

**Published:** 2017-08-08

**Authors:** Chiemi Imaizumi, Harumi Tomatsu, Kiminari Kitazawa, Yoshihisa Yoshimi, Seiji Shibano, Kaoru Kikuchi, Masatoshi Yamaguchi, Satoshi Kaneko, Yoichi Tsumuraya, Toshihisa Kotake

**Affiliations:** 1 Graduate School of Science and Engineering, Saitama University, Shimo-okubo, Sakura-ku, Saitama, Japan; 2 Faculty of Agriculture, University of the Ryukyus, Senbaru, Nishihara, Okinawa, Japan

**Keywords:** α-galactosidase, β-l-arabinopyranosidase, arabinogalactan-protein, cell wall, glycoside hydrolase 27 family, recombinant enzyme

## Abstract

The major plant sugar l-arabinose (l-Ara) has two different ring forms, l-arabinofuranose (l-Ara*f*) and l-arabinopyranose (l-Ara*p*). Although l-Ara mainly appears in the form of α-l-Ara*f* residues in cell wall components, such as pectic α-1,3:1,5-arabinan, arabinoxylan, and arabinogalactan-proteins (AGPs), lesser amounts of it can also be found as β-l-Ara*p* residues of AGPs. Even though AGPs are known to be rapidly metabolized, the enzymes acting on the β-l-Ara*p* residues remain to be identified. In the present study, four enzymes, which we call β-l-ARAPASE (APSE) and α-GALACTOSIDASE 1 (AGAL1), AGAL2, and AGAL3, are identified as those enzymes that are likely to be responsible for the hydrolysis of the β-l-Ara*p* residues in *Arabidopsis thaliana*. An Arabidopsis *apse-1* mutant showed significant reduction in β-l-arabinopyranosidase activity, and an *apse-1 agal3-1* double-mutant exhibited even less activity. The *apse-1* and the double-mutants both had more β-l-Ara*p* residues in the cell walls than wild-type plants. Recombinant APSE expressed in the yeast *Pichia pastoris* specifically hydrolyzed β-l-Ara*p* residues and released l-Ara from gum arabic and larch arabinogalactan. The recombinant AGAL3 also showed weak β-l-arabinopyranosidase activity beside its strong α-galactosidase activity. It appears that the β-l-Ara*p* residues of AGPs are hydrolysed mainly by APSE and partially by AGALs in Arabidopsis.

## Teaser

Through the analysis of mutants and recombinant enzymes expressed in *Pichia* yeast, an Arabidopsis β-l-arabinopyranosidase and α-d-galactosidases are shown to be responsible for the hydrolysis of β-l-arabinopyranosyl residues.

## Introduction

The physiologically important plant sugar l-arabinose (l-Ara) exists in both pyranose and furanose ring forms, which are respectively called l-arabinopyranose (l-Ara*p*; sugars other than l-Ara are in pyranose form unless stated otherwise) and l-arabinofuranose (l-Ara*f*). l-Ara mainly exists as α-l-arabinofuranosyl (α-l-Ara*f*) residues of cell wall polysaccharides and proteoglycans such as pectic α-1,3:1,5-arabinan (pectic arabinan), arabinoxylan, and arabinogalactan-proteins (AGPs). However, it can also be found in lesser amounts in the form of β-l-arabinopyranosyl (β-l-Ara*p*) residues in the AGPs of plants (Tryfona *et al.*, 2010; Kotake *et al.*, 2016), and as α-l-arabinopyranosyl (α-l-Ara*p*) residues in pectin rhamnogalacturonan II, xylan in the primary cell walls of monocots and some dicots, and β-l-arabinofuranosyl (β-l-Ara*f*) residues in glycan chains of extensin (Lamport *et al.*, 1973; McNeill *et al.*, 1984; Kieliszewski *et al.*, 1995; O’Neill *et al.*, 2001; Mohnen, 2008, Mortimer *et al.*, 2015, Peña *et al.*, 2016).

AGPs are a family of extracellular proteoglycans implicated in growth, cell differentiation, cell-to-cell signaling, cell adhesion, stress responses, pollen tube growth, and fertilization (Fincher *et al.*, 1983; Cheung *et al.*, 1995; Wu *et al.*, 1995; Majewska-Sawka and Nothnagel, 2000; van Hengel *et al.*, 2002; Shi *et al.*, 2003; van Hengel and Roberts, 2003; Motose *et al.*, 2004; Seifert and Roberts, 2007; Mizukami *et al.*, 2016). AGPs consist of hydroxyproline-rich core proteins and large arabinogalactan (AG) chains. The basic structure of the AG moiety, the so-called type II AG, comprises a β-1,3-galactan main chain and β-1,6-galactan side chains. The side chains are substituted with auxiliary sugars such as l-Ara*f*, glucuronic acid (GlcA – the d-prefix is omitted for sugars belonging to the d-series), and 4-*O*-methyl-GlcA (4-Me-GlcA) (Tsumuraya *et al.*, 1987, 1988; Tan *et al.*, 2010). In several plants, including wheat (*Triticum aestivum*), larch (*Larix dahurica*), and Scots pine (*Pinus sylvestris*), β-l-Ara*p* residues can be found as non-reducing terminal residues joined to α-l-Ara*f* residues through 1,3-linkages (Odonmazig *et al.*, 1994; Ponder and Richards, 1997; Willför *et al.*, 2002; Tryfona *et al.*, 2010), although they are less abundant than the β-Gal, α-l-Ara*f*, GlcA, and 4-Me-β-GlcA residues found in the AGPs of many other plants (Tan *et al.*, 2010; Tryfona *et al.*, 2012; Shimoda *et al.*, 2014). Mung bean hypocotyls exhibit β-1,3-arabinopyranosyltransferase activity, probably catalysing the synthesis of the β-l-Ara*p* residues (Ishii *et al.*, 2005).

The structures of the AG moieties differ depending on tissue, organ, and plant (Tsumuraya *et al.*, 1987, 1988). It is highly probable that the specific structures are determined by glycoside hydrolases (GHs) trimming and degrading the AG moieties, and do not solely depend on the glycosyltransferases that are involved in the synthesis. Indeed, extremely rapid turnover of the AG moieties has been reported in proso millet cells (Gibeaut and Carpita, 1991). As the carbohydrate moieties of AGPs consist of many types of sugar residues, such as β-Gal, α-l-Ara*f*, and 4-Me-β-GlcA, various GHs participate in the hydrolysis (Knoch *et al.*, 2014). Land plants have β-galactosidases that prefer β-1,3- and β-1,6-Gal residues in type II AG to β-1,4-Gal residues in pectic β-1,4-galactan (Kotake *et al.*, 2005; Eda *et al.*, 2014, 2016). On the basis of amino acid sequence and structural similarity, these β-galactosidases are categorized as members of the GH35 family (Henrissat, 1991; Henrissat and Bairoch, 1993).

Because the hydrolysis of β-1,3:1,6-galactan by β-galactosidases never occurs in an endo-manner in plants, it is highly probable that non-reducing terminal residues, such as α-l-Ara*f*, β-l-Ara*p*, and β-GlcA, must be hydrolysed first. In other words, the hydrolysis of these auxiliary sugar residues partially regulates the degradation and metabolism of the AG moieties. Several GHs hydrolysing the auxiliary sugar residues have been identified. It is known that α-l-arabinofuranosidases from the GH3 family act on non-reducing terminal α-l-Ara*f* residues of AG moieties (Hata *et al.*, 1992; Kotake *et al.*, 2006). The non-reducing terminal β-GlcA and 4-Me-β-GlcA residues are presumably hydrolysed by endogenous β-glucuronidase(s), designated AtGUS1, 2, and/or 3, belonging to the GH79 family, as the overexpression of *AtGUS2* reduces these residues in AGPs in Arabidopsis (*Arabidopsis thaliana*) (Eudes *et al.*, 2008). This interpretation of the function of these AtGUSs is also supported by significant sequence similarity to fungal β-glucuronidases belonging to the GH79 family that are active on β-GlcA and 4-Me-β-GlcA residues of AGP (Konishi *et al.*, 2008; Michikawa *et al.*, 2012). As well as α-l-Ara*f* and β-GlcA residues, β-l-Ara*p* residues are also probably removed prior to the hydrolysis of β-1,3:1,6-galactan in the metabolism of AG moieties. Although an enzyme with activity toward *p*-nitrophenyl-β-l-arabinopyranoside (PNP-β-l-Ara*p*) has previously been detected and purified from pigeon pea (*Cajanus indicus*) (Dey, 1973, 1983), its properties, including its action on natural substrates, remain to be determined.

**Fig. 1.**
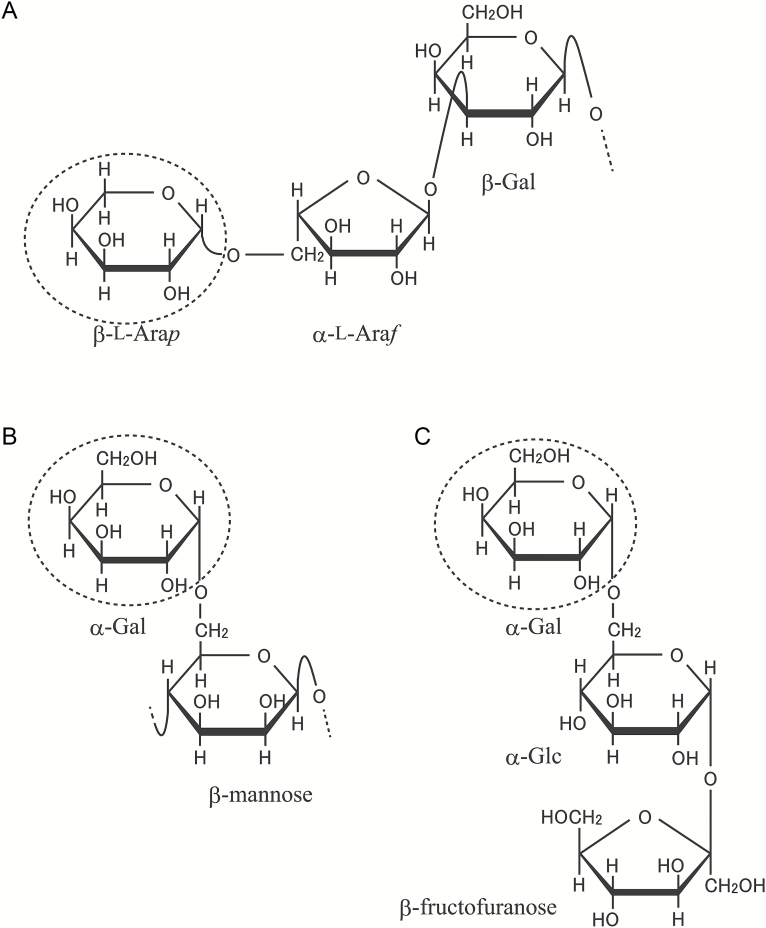
Partial structures of β-l-Ara*p*- and α-Gal-containing molecules. (A) Type II AG, (B) galactomannan, and (C) raffinose. The β-l-Ara*p* and α-Gal residues are ringed by dashed lines. Sugars are drawn using a Haworth projection.

AG moieties also undergo active hydrolysis by bacteria and fungi in nature. Several β-l-arabinopyranosidases hydrolysing β-l-Ara*p* residues of AGP have been found and characterized in *Streptomyces avermitilis*, *Fusarium oxysporum*, *Geobacillus stearothermophilus*, and *Chitinophaga pinensis* (Ichinose *et al.*, 2009; Sakamoto *et al.*, 2010; Salama *et al.*, 2012; McKee and Brumer, 2015). These microbial β-l-arabinopyranosidases belong to the GH27 family and have amino acid sequences similar to those of α-galactosidases in this family, because the β-l-Ara*p* residue and the α-Gal residue are structurally similar (Ichinose *et al.*, 2009; Kotake *et al.*, 2016) (Fig. 1).

For the present study, four members of the GH27 family, possibly responsible for the hydrolysis of the β-l-Ara*p* residues in Arabidopsis, were identified. On the basis of the phenotypes of Arabidopsis mutants with defects in these enzymes and the properties of recombinant enzymes expressed in the yeast *Pichia pastoris*, one of them, designated β-l-ARAPASE (APSE), appeared to be a β-l-arabinopyranosidase specifically acting on β-l-Ara*p* residues of AGP. In addition, one α-galactosidase, designated α-GALACTOSIDASE 3 (AGAL3), was found to have weak β-l-arabinopyranosidase activity and may participate in the hydrolysis of the β-l-Ara*p* residues in Arabidopsis.

## Materials and methods

### Plant materials

*Arabidopsis thaliana* ecotype Columbia (Col) was used in this study. The T-DNA insertion lines, SALK_016141 (*apse-1*), SALK_083934 (*agal1-1*), SALK_134497 (*agal2-1*), and SALK_012366C (*agal3-1*), were provided by the Arabidopsis Biological Resource Center (The Ohio State University, USA; Supplementary Fig. S1 at *JXB* online). The *apse-1 agal3-1* double-mutant was generated by a cross between homozygous *apse-1* and homozygous *agal3-1*. The genotypes of the mutants were determined using specific primers (Supplementary Table S1). The Arabidopsis seedlings were germinated and grown on Murashige–Skoog (MS) medium in 0.8% (w/v) agar under continuous light at 23 °C for 2 weeks, then on rock wool fibers (Nittobo, Tokyo, Japan) under the same conditions (Murashige and Skoog, 1962).

For overexpression of *APSE* and *AGAL3*, the cDNAs were amplified using the sets of primers shown in Supplementary Table S1 and cloned between the *Bam*HI site and *Sac*I site of pBI121 (Clontech, Palo Alto, CA, USA). The plasmids were introduced into Arabidopsis (ecotype Columbia) by an Agrobacterium- (*Rhizobium radiobacter*, EHA105 strain) mediated method (Clough and Bent, 1998). For the analysis of enzyme activity, T2 populations of the *APSE*-overexpressing (*APSE*-OX) line 2 (#2) and *AGAL3*-overexpressing (*AGAL*-OX) #7 were used. The transgenic plants including homozygous or heterozygous transgenes were selected based on the resistance to kanamycin.

### Oligo- and polysaccharides

The following were purchased from Sigma (St. Louis, MO, USA): PNP-α-l-arabinofuranoside (PNP-α-l-Ara*f*), PNP-β-l-Ara*p*, PNP-α-l-fucoside (PNP-α-l-Fuc), PNP-α-galactoside (PNP-α-Gal), PNP-β-galactoside (PNP-β-Gal), PNP-α-glucoside (PNP-α-Glc), PNP-β-glucoside (PNP-β-Glc), PNP-β-glucuronide (PNP-β-GlcA), guar gum (galactomannan from *Cyamopsis tetragonoloba*), gum arabic from acacia (*Acacia senegal*), locust bean gum (galactomannan from *Ceratonia siliqua*), laminarin (β-1,3:1,6-glucan) from *Laminaria digitate*, and β-1,4-xylan from birchwood. β-1,4-Galactan from lupin (*Lupinus albus*) was from Megazyme (Wicklow, Ireland). Larch wood (*Larix laricina*) AG was from Tokyo Kasei (Tokyo, Japan).

### Analytical methods

The concentration of protein was determined by the method of Bradford (1976) with bovine serum albumin as the standard. Reducing sugars were estimated by the method of Dygert *et al.* (1965). Total sugars were determined by the phenol-sulfuric acid method (Dubois *et al.*, 1956). Sugar composition analysis was carried out by high-performance anion-exchange chromatography (HPAEC) with pulsed amperometric detection (PAD) using a Dionex DX-500 liquid chromatograph fitted with a CarboPac PA-1 column and a pulsed amperometric detector, as described previously (Ishikawa *et al.*, 2000).

### Phylogenetic analysis

The amino acid sequences of GH27 enzymes were collected at NCBI BLAST (blast.ncbi.nlm.nih.gov/Blast.cgi). The alignments of amino acid sequences were performed using the MUSCLE program in the MEGA software package (version 6; Tamura *et al.*, 2013). Phylogenetic trees were generated by the neighbor-joining method with 1000 bootstrap replications. The accession numbers and abbreviations used are listed in Supplementary Table S2.

### Preparation of recombinant enzymes

Total RNA was extracted from 2-week-old Arabidopsis seedlings with an Isogen kit (Nippon Gene, Tokyo, Japan) according to the manufacturer’s instructions. Single-strand cDNA was synthesized from 1 µg of total RNA from the seedlings using a reverse-transcriptase, ReverTra Ace-α- (Toyobo, Osaka, Japan) and oligo(dT)_12–18_ primer (Invitrogen, Carlsbad, CA, USA). The cDNA fragments corresponding to Arg33-Ala647 of APSE (At3g26380.1), Phe26-Ala396 of α-GALACTOSIDASE 2 (AGAL2, At5g08370.1), and Gly31-Val437 of AGAL3 (At3g56310.1) were amplified using sets of specific primers listed in Supplementary Table S1. The cDNA fragments were subcloned between the *Eco*RI and *Spe*I sites that are preceded by yeast α-factor of pPICZαC (Invitrogen). The methylotrophic yeast *P. pastoris* strain KM71 was transformed with the linearized plasmid construct with a multicopy *Pichia* expression kit (Invitrogen). The transformants resistant to zeocin were screened according to the manufacturer’s instructions.

The transformants were cultured in medium containing 1% (w/v) yeast extract, 2% (w/v) peptone, and 1% (w/v) glycerol at 28 °C with shaking at 100 rpm for 2 d. The cells were harvested by centrifugation, washed with ice-cold distilled water, then suspended in 50 ml of the medium containing 1% (w/v) yeast extract, 2% (w/v) peptone, and 1% (v/v) methanol. The yeast cells were cultured for another 5 d at 28 °C, during which time 0.5 ml of methanol was added each day, to induce the recombinant enzyme.

The culture medium of the *Pichia* cells was centrifuged, and the supernatant was collected. The sample was desalted with a PD-10 column (GE Healthcare, Tokyo, Japan) for recombinant APSE (rAPSE) or dialysed against 20 mM sodium acetate buffer (pH 5.0) for rAGAL2 and rAGAL3, and passed through a TOYOPEARL HW65 (Tosoh, Tokyo, Japan) column to remove cell debris. rAPSE was purified by cation-exchange chromatography using a CM-Sepharose Fast Flow (GE Healthcare) column. rAGAL2 was purified by cation-exchange chromatography using CM-Sepharose Fast Flow and then by gel-permeation chromatography using a Sephacryl S-200 (GE Healthcare) column. rAGAL3 was purified by anion-exchange chromatography using a DEAE-Sepharose Fast Flow (GE Healthcare) column and then by gel-permeation chromatography using Sephacryl S-200. The purity of the recombinant enzymes was determined on sodium dodecyl sulfate-polyacrylamide gel electrophoresis (SDS-PAGE) (Laemmli, 1970).

### Enzyme assays

The β-l-arabinopyranosidase activity of recombinant proteins was determined using a reaction mixture (100 μl) consisting of the enzyme, 1 mM PNP-β-l-Ara*p*, and 50 mM sodium acetate buffer (pH 4.0 for rAPSE, pH 5.5 for rAGALs). After incubation at 37 °C for the appropriate reaction time, the reaction was terminated by addition of 200 mM Na_2_CO_3_ (900 μl) and monitored at 420 nm. One unit of enzyme activity liberates 1 µmole of *p*-nitrophenol per minute.

For the determination of the substrate specificity toward poly- and oligosaccharides, enzyme activity was measured using reaction mixtures (100 µl) consisting of the enzyme, 0.25% (w/v) poly- or oligosaccharide, and 50 mM sodium acetate buffer (pH 4.0 for rAPSE, pH 5.5 for rAGALs). After incubation at 37 °C, the liberated sugars were detected and quantified by HPAEC-PAD.

### Measurement of enzyme activity in plants

Arabidopsis plants grown for 35 d on rock wool fibers were homogenized with mortar and pestle in 20 mM sodium acetate buffer (pH 5.0). After centrifugation at 10 000 *g* for 5 min, the supernatant was collected (the soluble fraction). The precipitate (the cell wall fraction) was washed once with the same buffer and then suspended in the buffer with addition of 1 M NaCl to release cell wall-bound protein. After centrifugation at 10 000 *g* for 5 min, the supernatant was collected (the cell wall-bound fraction). The β-l-arabinopyranosidase activity was measured using a reaction mixture (100 μl) consisting of the soluble or cell wall-bound fraction, 1 mM PNP-β-l-Ara*p*, and 50 mM sodium acetate buffer (pH 5.0) at 37 °C.

### Quantification of l-Ara*p* residues in cell walls

The cell wall fraction prepared as described above was used. The cell wall was treated with microbial β-l-arabinopyranosidase (SaArap27A; obtained in our previous study; Ichinose *et al.*, 2009) at 37 °C for 24 h to release l-Ara*p* residues remaining in the cell walls. The released l-Ara was measured by HPAEC-PAD.

## Results

### GH27 enzyme genes in Arabidopsis

Although plant β-l-arabinopyranosidase remains to be identified, several microbial β-l-arabinopyranosidases belonging to the GH27 family have been reported (Ichinose *et al.*, 2009; Sakamoto *et al.*, 2010; Salama *et al.*, 2012; McKee and Brumer, 2015). These β-l-arabinopyranosidases share amino acid sequences similar to those of α-galactosidases in the GH27 family, as the structure of β-l-Ara*p* is similar to that of α-Gal (Fig. 1). The genome of Arabidopsis includes four genes for GH27 family enzymes. Among them, two proteins (At5g08380.1 and At5g08370.1) have been characterized as α-GALACTOSIDASE 1 (AGAL1) and AGAL2 with activity toward α-Gal residues of raffinose oligosaccharides (Tapernoux-Lüthi *et al.*, 2004). In the present study, together with AGAL1 and AGAL2, two other proteins (At3g26380.1 and At3g56310.1), APSE and AGAL3, respectively, were identified as candidate enzymes responsible for the hydrolysis of β-l-Ara*p* residues in Arabidopsis. In contrast to α-galactosidases belonging to GH family 36, such as AtSIP2 in Arabidopsis (Peters *et al.*, 2010), AGALs and APSE have a signal peptide at the N-terminus, suggesting that these enzymes are secreted.

**Fig. 2.**
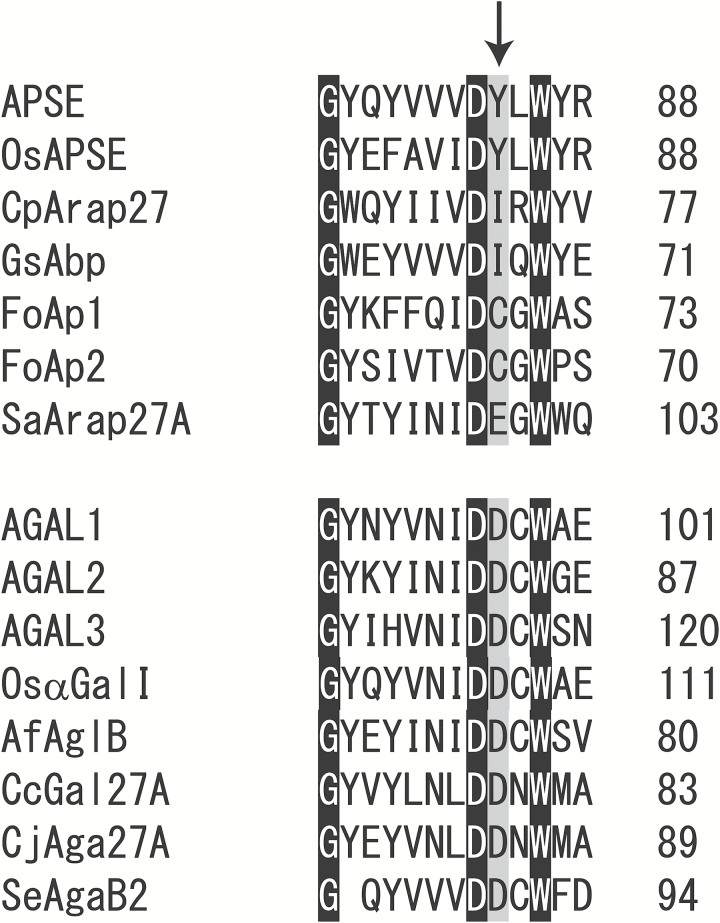
Amino acid residues important for substrate specificity. Partial amino acid sequences of GH27 enzymes were aligned by the pairwise method using the ClustalW program. The amino acid residue corresponding to Glu99 in SaArap27A involved in the recognition of the α-Gal or β-l-Ara*p* residue is indicated by an arrow. As in microbial β-l-arabinopyranosidases, there is a bulky amino acid residue, Tyr, at this point in APSE and OsAPSE, whereas AGALs tend to have Asp, which is conserved for many α-galactosidases in the GH27 family. Residues conserved in all proteins are highlighted in black.

**Fig. 3.**
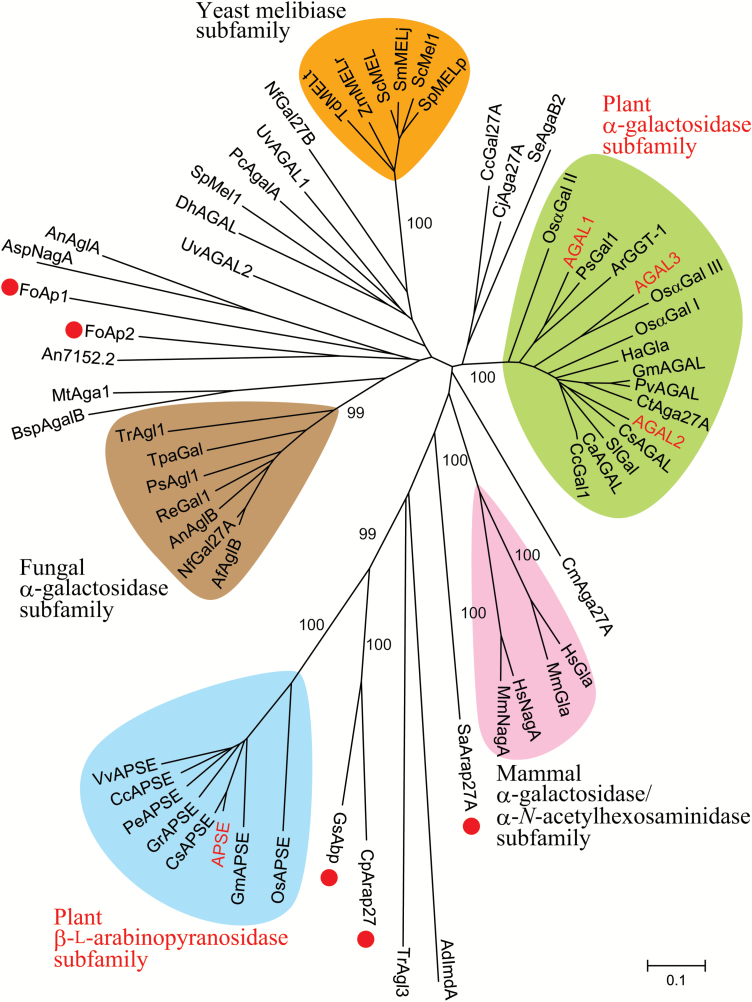
The phylogenetic relationships of β-l-arabinopyranosidases and α-galactosidases belonging to the GH27 family. β-l-Arabinopyranosidases are marked with red circles. The analysis was performed using MEGA software (version 6.0, Tamura *et al.*, 2013). The scale bar indicates substitutions per site. Numbers at nodes represent bootstrap values. The accession numbers for amino acid sequences are listed in Supplementary Table S1.

In our previous studies, an amino acid residue in GH27 family enzymes was shown to be involved in the recognition of β-l-Ara*p* and α-Gal residues (Fujimoto *et al.*, 2003; Ichinose *et al.*, 2009). A rice α-galactosidase, OsαGal II, with Asp in the position prefers α-Gal residues as its substrate. This Asp residue is highly conserved in many GH27 α-galactosidases, whereas it is replaced with bulkier amino acids such as Glu and Ile in microbial β-l-arabinopyranosidases (Fig. 2) (Ichinose *et al.*, 2009; Salama *et al.*, 2012; McKee and Brumer, 2015). Among the four GH27 enzymes in Arabidopsis, only APSE has a bulky residue, Tyr, at the corresponding position. Therefore, APSE was presumed to be a β-l-arabinopyranosidase, whereas AGAL3, with the Asp residue in this position, was viewed, like AGAL1 and AGAL2, as an α-galactosidase. In the phylogenetic tree, APSE was located near a microbial β-l-arabinopyranosidase (GsAbp from *Geobacillus stearothermophilus*) together with close homologues in plants (Fig. 3, Supplementary Table S2). On the other hand, AGAL1, AGAL2, and AGAL3 were grouped into the plant α-galactosidase subfamily with known plant α-galactosidases including OsαGal II (Fujimoto *et al.*, 2003; Li *et al.*, 2007).

### Decreased *β*-l-arabinopyranosidase activity in the *apse-1* mutant

**Fig. 4.**
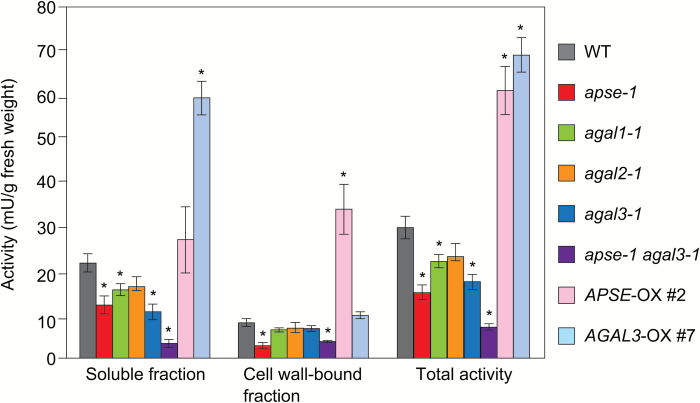
β-l-Arabinopyranosidase activity in the mutants and in *APSE*- and *AGAL3*-OX plants. The soluble and cell wall-bound fractions were prepared from Arabidopsis seedlings grown for 2 weeks on MS-agar medium. Data are mean values with ±SE (*n*=3 biological replicates). Asterisks indicate significant differences from the wild-type (WT) plants (Student’s *t*-test, *P*<0.05). The activities of *APSE*-OX #2 and *AGAL3*-OX #7 are shown here: results for the other *APSE*- and *AGAL3*-OX lines are shown in Supplementary Fig. S2.

To examine the participation of APSE and AGALs in the hydrolysis of β-l-Ara*p* residues *in vivo*, β-l-arabinopyranosidase activity in the seedlings of *apse-1*, *agal1-1*, *agal2-1*, and *agal3-1* mutants was measured. It is probable that the AGALs also have β-l-arabinopyranosidase activity as well as α-galactosidase activity, because the structures of β-l-Ara*p* and α-Gal are similar (Fig. 1). In the present study, β-l-arabinopyranosidase activity in the soluble fraction obtained as the supernatant of the homogenate and the cell wall-bound fraction extracted from the residual precipitate with 1 M NaCl were measured using PNP-β-l-Ara*p* as the substrate. As shown in Fig. 4, the *apse-1* mutant showed a significant reduction in the activity mainly in the cell wall-bound fraction compared with wild-type (WT) plants. The total activity in *apse-1* was approximately 50% of the WT. The *agal3-1* mutants showed approximately 30% reduction in total activity. In addition, the *apse-1 agal3-1* double-mutant exhibited further reduction in activity compared with the *apse-1* mutant. These results indicate that β-l-arabinopyranosidase activity in the seedlings can be mainly attributed to APSE and AGAL3. The reduction of the activity in the a*gal2-1* mutant was not obvious compared with *agal3-1*. According to expression data obtained from Genevestigator (Hruz *et al.*, 2008), *AGAL3* has a higher expression level than *AGAL2*. Hence, the *agal3-1* mutation probably affected the total β-l-arabinopyranosidase activity more than the *agal2-1* mutation (Supplementary Fig. S4).

### Effect of overexpression of *APSE* and *AGAL3* on *β*-l-arabinopyranosidase activity

The β-l-arabinopyranosidase activity of APSE and AGAL3 was confirmed by overexpression of *APSE* and *AGAL3* genes. As shown in Fig. 4 and Supplementary Fig. S2, the *APSE*-OX plants exhibited higher β-l-arabinopyranosidase activity than WT plants in terms of the total activity. Because the activity was quite high in the cell wall-bound fraction compared with that in the soluble fraction, we suggest that APSE probably binds to cell wall components. The overexpression of *AGAL3* also significantly raised β-l-arabinopyranosidase activity, although higher activity was mainly observed in the soluble fraction, suggesting that AGAL3 is secreted but not tightly bound to cell wall components. These results strongly support the hypothesis that APSE and AGAL3 function as enzymes with β-l-arabinopyranosidase activity in Arabidopsis.

### Increased *β*-l-Ara*p* residues in cell walls in the *apse-1* mutant

**Fig. 5.**
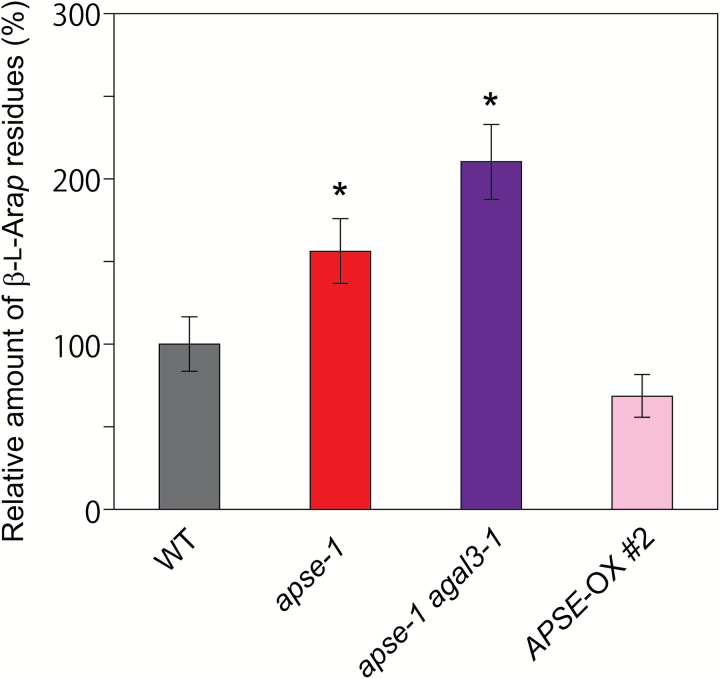
Amount of β-l-Ara*p* residues remaining in the cell walls. β-l-Ara*p* released from the cell wall fraction by SaArap27A, which is known to act effectively on residues in AGPs, was measured by HPAEC-PAD. Data are mean values ±SE (*n*=3 biological replicates). Asterisks indicate a significant difference from the wild-type (WT) plants (Student’s *t*-test, *P*<0.05).

If β-l-Ara*p* residues are mainly hydrolysed by APSE, more β-l-Ara*p* residues should remain in the *apse-1* mutant. The amount of β-l-Ara*p* residues in the AGP-rich fraction prepared from cell walls was therefore measured. In this experiment, remaining β-l-Ara*p* residues were released using SaArap27A, which effectively acts on β-l-Ara*p* residues, and were quantified by HPEAC-PAD (Ichinose *et al.*, 2009). The amount of remaining β-l-Ara*p* residues was significantly higher in *apse-1* and the double-mutants compared with the WT (Fig. 5), indicating that the β-l-Ara*p* residues are indeed mainly hydrolysed by APSE.

### Properties of rAPSE, rAGAL2, and rAGAL3

**Fig. 6.**
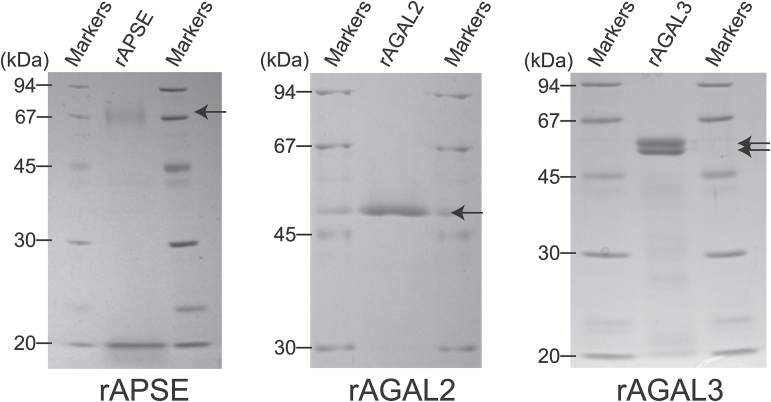
SDS-PAGE of recombinant enzymes. AGAL2, rAGAL3, and rAPSE were prepared by heterologous expression in *P. pastoris* and purified by conventional chromatography. Proteins in the gel were stained with Coomassie Brilliant Blue R-250. The arrows indicate the purified recombinant enzymes.

In order to understand the properties of APSE and AGALs, rAPSE, rAGAL2, and rAGAL3 were prepared. Based on the SignalP 3.0 program for the prediction of signal sequences, APSE, AGAL2, and AGAL3 have a signal sequence (Bendtsen *et al.*, 2004). Hence, each cDNA – but excluding the sequence corresponding to the signal peptides – was isolated by RT-PCR, subcloned to an expression vector, and introduced into the methylotrophic yeast *P. pastoris*. rAPSE, rAGAL2, and rAGAL3 were purified by conventional chromatography (Supplementary Tables S3–S5). As the yield of rAPSE was quite low compared to rAGAL2 and rAGAL3, we suspect that rAPSE may be unstable in *P. pastoris* cells. The purity of the recombinant enzymes was confirmed on SDS-PAGE (Fig. 6). The apparent sizes observed for rAPSE (72 kDa), rAGAL2 (46 kDa), and rAGAL3 (57 and 53 kDa) on SDS-PAGE were slightly larger than those calculated based on the deduced amino acid sequences (APSE, 68 863 Da; AGAL2, 41 282 Da; AGAL3, 45 096 Da), which may be explained by *N*-glycosylation(s). Indeed, NetNGlyc 1.0 (www.cbs.dtu.dk/services/NetNGlyc/, Joshi and Gupta, 2015), predicts that APSE, AGAL2, and AGAL3 have two possible *N*-glycosylation sites each. The two protein bands of rAGAL3 presumably derive from variations in the *N*-glycosylation in *P. pastoris* cells.

The basic properties of rAPSE were examined using PNP-β-l-Ara*p* as the substrate, and those of rAGAL2 and rAGAL3 using PNP-α-Gal. rAPSE showed maximum activity at pH 4, whereas rAGAL2 and rAGAL3 were most active at pH 5.5 (Supplementary Fig. S3).

### Substrate specificity of the recombinant enzymes

The substrate specificity of rAPSE, rAGAL2, and rAGAL3 toward sugar residues was examined using various PNP-substrates. rAPSE specifically acted on PNP-β-l-Ara*p* (Table 1). Importantly, the strong α-galactosidase activity of rAGAL2 and rAGAL3 was accompanied by weak β-l-arabinopyranosidase activity. The results strongly suggest that not only APSE but also AGAL2 and AGAL3 participate in the hydrolysis of β-l-Ara*p* residues in Arabidopsis.

**Table 1.** Substrate specificity of recombinant enzymes toward PNP-sugars

PNP-Sugar^a^	rAPSE^b^	rAGAL2^c^	rAGAL3^c^
PNP-α-l-Ara*f*	<0.1	0.2	<0.1
PNP-β-l-Ara*p*	100.0	10.6	7.0
PNP-α-l-Fuc	<0.1	<0.1	<0.1
PNP-α-Gal	<0.1	100.0	100.0
PNP-β-Gal	<0.1	<0.1	<0.1
PNP-α-Glc	<0.1	<0.1	<0.1
PNP-β-Glc	<0.1	<0.1	<0.1
PNP-β-GlcA	<0.1	<0.1	<0.1

^a^The enzymes were incubated with 1 mM PNP-sugars.

^b^Activity is expressed as % of that toward PNP-β-l-Ara*p*.

^c^Activity is expressed as % of that toward PNP-α-Gal.

### Action on natural substrates

Larch wood AG and gum arabic have large (3.9%) and small amounts (1.9%) of β-l-Ara*p* residues, respectively (Akiyama *et al.*, 1984; Ponder and Richards, 1997; Willför *et al.*, 2002). However, the β-l-Ara*p* residue is absent or very rare in AGPs from radish roots and leaves (Tsumuraya *et al.*, 1987, 1988). The activity of rAPSE and rAGALs toward these AG and AGPs was also examined (Table 2). rAPSE substantially acted on larch wood AG, releasing free l-Ara. rAGAL2 and rAGAL3 also released l-Ara, but the amount was low compared with rAPSE. As rAGAL2 has weak α-l-arabinofuranosidase activity as well as α-galactosidase and β-l-arabinopyranosidase activities (Table 1), it is possible that rAGAL2 acted on α-l-Ara*f* residues, rather than β-l-Ara*p* residues. Because released l-Ara*f* may spontaneously convert to more stable l-Ara*p*, we were not able to determine which sugar was hydrolysed. rAGAL3 substantially released Gal from guar gum and locust bean gum, in which Gal residues occupy approximately 30% and 20% of the weight, respectively, suggesting that the main substrate for AGAL3 is galactomannan or galactoglucomannan (Daas *et al.*, 2000).

**Table 2.** Actions of recombinant enzymes on natural substrates

Substrate^a^	Limit of hydrolysis (%)^a^		
	rAPSE	rAGAL2	rAGAL3
AGP from radish roots^b^	<0.1	<0.1	0.1
AGP from acacia (gum arabic)^b^	6.0	0.1	0.7
AG from larch wood^b^	10.6	0.5	2.9
Galactomannan from locust bean^c^	<0.1	<0.1	<0.1
Galactomannan from guar^c^	<0.1	<0.1	<0.1
β-1,4-Xylan	<0.1	<0.1	<0.1
Pectic galactan^c^	0.2	0.8	0.2
β-1,3:1,4-Glucan	<0.1	<0.1	<0.1

^a^The reaction was carried out using 1 mUnit of recombinant enzyme and 2.5 mg ml^–1^ polysaccharide substrate at 37 °C for 24 h. Released l-Ara was detected and quantified by HPAEC-PAD.

^b^Proportion (%) of released l-Ara to total l-Ara residues included in the substrate.

^c^Proportion (%) of released Gal to total Gal residues included in the substrate.

### Growth of hypocotyls of mutants

**Fig. 7.**
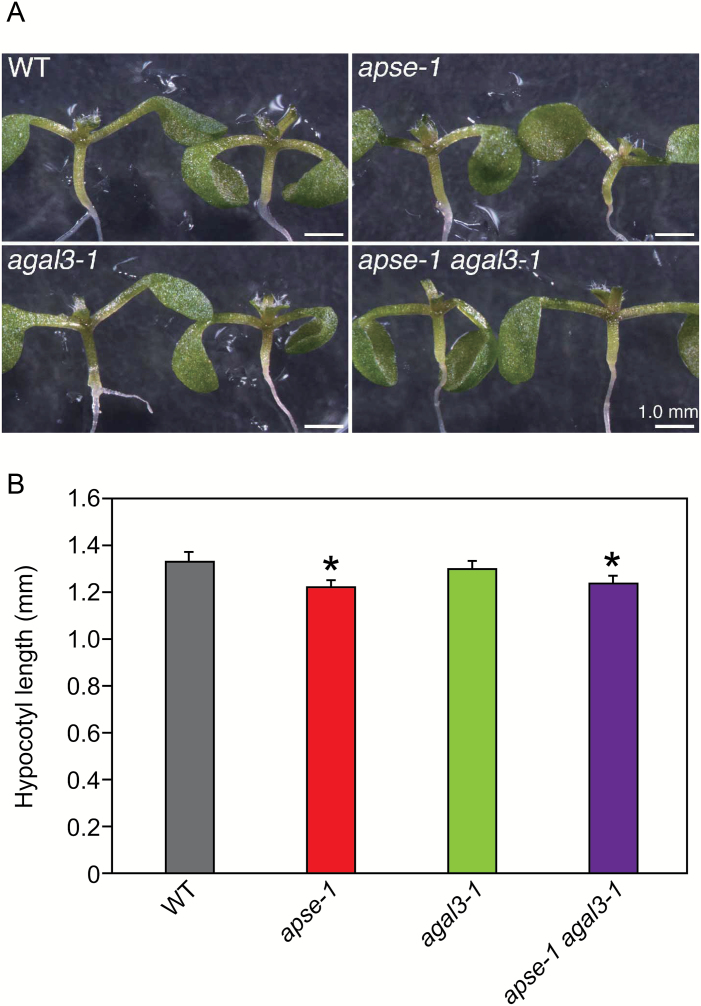
Growth of hypocotyls. (A) Representative pictures of seedlings of the wild-type (WT), *apse-1*, *agal3-1*, and the double-mutants. Scale bars indicate 1.0 mm. To show the hypocotyls clearly, some main leaves were excised. (B) Hypocotyl lengths of seedlings grown on MS-agar medium for 7 d under continuous light. Data are mean values ±SE (*n*=10 biological replicates). Asterisks indicate significant differences from WT plants (Student’s *t*-test, *P*<0.05).

The growth of hypocotyls of *apse* and *agal* mutants were examined. The *apse-1* and the double-mutants showed significantly shorter hypocotyls than the WT plants (Fig. 7). Because the hypocotyls of mutants did not reach the length of those of WT plants even after extended incubation (data not shown), we conclude that the shorter hypocotyl is caused by reduced growth, rather than a delay of germination. The mutants did not show any other phenotype significantly different from the WT plants.

## Discussion

### Plant GH27 enzymes acting on β-l-Ara*p* residues

AG moieties undergo rapid hydrolysis *in vivo* (Gibeaut and Carpita, 1991) and to date, β-galactosidase, α-l-arabinofuranosidase, and β-glucuronidase have been identified as enzymes involved (Kotake *et al.*, 2005, 2006; Eudes *et al.*, 2008; Eda *et al.*, 2014). The present study has identified APSE as a plant β-l-arabinopyranosidase participating in the hydrolysis of AG moieties of AGPs. The wide distribution of genes with high similarity to APSE in land plants suggests that β-l-arabinopyranosidases are conserved and physiologically important. It is worth mentioning that pigeon pea (*Cajanus cajan*) has a gene encoding a GH27 protein (accession number XP_020221274.1) highly related to APSE in its genome (Dey, 1973, 1983). On the basis of reduced β-l-arabinopyranosidase activity in *agal* mutants and weak but significant β-l-arabinopyranosidase activity (as well as strong α-galactosidase activity) of rAGAL2 and rAGAL3, AGALs can also be assumed to hydrolyze β-l-Ara*p* residues in AGPs. Like other non-reducing terminal residues including α-l-Ara*f* and β-GlcA residues, β-l-Ara*p* residues probably affect the hydrolysis of the β-1,3:1,6-galactan, because plant β-galactosidases act on the galactan in an exo-manner (Kotake *et al.*, 2005; Eda *et al.*, 2014). Therefore, it seems likely that APSE and AGALs affect the metabolism of AGP both directly and indirectly. It is conceivable that APSE and AGALs act on different β-l-Ara*p* residues in AGPs: we observed that the recombinant enzymes have different limits of hydrolysis for both gum arabic and larch AG (Table 2).

### Substrate specificity of GH27 β-l-arabinopyranosidase and α-galactosidase

In plant GH27 α-galactosidases, a conserved Asp residue in the catalytic pocket provides the space for the C-6 hydroxylmethyl group of α-Gal. This Asp residue is replaced with bulkier residues such as Glu and Ile at the corresponding site in GH27 β-l-arabinopyranosidases (Fujimoto *et al.*, 2003; Li *et al.*, 2007; Ichinose *et al.*, 2009; Salama *et al.*, 2012; McKee and Brumer, 2015). The bulkier residues can be presumed to change the interaction of the enzyme with α-Gal residues, which affects the substrate specificity toward β-l-Ara*p* and α-Gal residues. The results in the present study substantially support the importance of the residue for the recognition of β-l-Ara*p* and α-Gal residues. In fact, while rAGAL2 and rAGAL3 with the conserved Asp residue preferred α-Gal to β-l-Ara*p* residues, rAPSE with Tyr at the corresponding position specifically acted on β-l-Ara*p* residues. However, in many GH27 β-l-arabinopyranosidases and α-galactosidases – including SaArap27A, FoAp1, and FoAp2 from *Fusarium oxysporum* and AGALs – the substrate specificity is not completely determined by this residue (Ichinose *et al.*, 2009; Sakamoto *et al.*, 2010). These facts suggest that there are other amino acid residues affecting the substrate specificity toward β-l-Ara*p* and α-Gal residues.

### Substrates for APSE and AGALs

It has been reported that several AGPs and type II AGs have β-l-Ara*p* residues in their carbohydrate moieties (Odonmazig *et al.*, 1994; Ponder and Richards, 1997; Willför *et al.*, 2002; Tryfona *et al.*, 2010). However, there may be β-l-Ara*p*-containing molecules serving as substrates for APSE and AGALs other than these AGPs and type II AGs. In fact, β-l-Ara*p* residues are sometimes seen in other cell wall components. For instance, β-l-Ara*p* exists as a non-reducing terminal residue in pectic α-1,3:1,5-arabinan in the common marsh mallow (*Althaea officinalis*) and pigeon pea (*C. cajan*) (Capek *et al.*, 1983; Swamy and Salimath, 1991). β-l-Ara*p* also constitutes the non-reducing terminus of type I AG of soybean (*Glycine max*) (Huisman *et al.*, 2001). Therefore, we cannot exclude the possibility that APSE and AGALs also contribute to the hydrolysis of β-l-Ara*p* residues of pectic α-1,3:1,5-arabinan and type I AG.

The rAGALs showed α-galactosidase activity toward raffinose and stachyose (Supplementary Table S6), but these oligosaccharides are not expected to be their main substrates *in vivo*, because, unlike GH36 enzymes such as AtSIP2 acting on α-Gal residues of raffinose and stachyose, AGALs have a signal peptide that causes them to localize to cell walls, suggesting that AGALs act on cell wall components with α-Gal and β-l-Ara*p* residues. To explore the *in vivo* substrate for AGALs, α-galactosidase activity of rAGALs was examined on seed mucilage and cell wall fractions prepared from different tissues (Supplementary Table S7). As shown in Supplementary Fig. S5, the rAGALs released Gal from seed mucilage, indicating that AGALs act on a polysaccharide included in the mucilage. Importantly, it has been reported that Arabidopsis synthesizes galactoglucomannan as a component of seed mucilage, although this polysaccharide is either not present or is rare in other tissues (Yu *et al.*, 2014; Voiniciuc *et al.*, 2015). Based on the data for gene expression patterns (Winter *et al.*, 2007), *AGAL2* and *AGAL3* are highly expressed in imbibed seeds (Supplementary Fig. S6). These facts suggest that one of the *in vivo* substrates for AGALs is galactoglucomannan included in the mucilage.

### Contribution of APSE to the growth of hypocotyls

The *apse-1* mutation significantly inhibited elongation growth of hypocotyls (Fig. 7), an observation reminiscent of a report that the loss of the *AtGUS* gene, which encodes a β-glucuronidase acting on GlcA residues of AGPs, also reduced elongation (Eudes *et al.*, 2008). These observations suggest that the hydrolysis of non-reducing terminal sugar residues of AGPs affects hypocotyl growth by changing AGP metabolism. Rapid hydrolysis of AG moieties might be required for the formation and modification of cell walls, as monomer l-Ara and Gal released from AGPs are recycled to respective UDP-sugars that serve as donor substrates in the synthesis of cell wall polysaccharides (Bar-Peled and O’Neill, 2011). It has also been proposed that AGPs render cell wall components soluble in the Golgi apparatus and secretion vesicles until they arrive at their proper sites at the cell surface (Gibeaut and Carpita, 1991). AGPs may have to be degraded immediately after they have performed their function. The physiological importance of the rapid metabolism of AGPs in plants should be further clarified in future studies.

## Supplementary Data

Supplementary data are available at *JXB* online.

Table S1. Sequences of primers used in the present study.

Table S2. Accession numbers for amino acid sequences.

Table S3. Purification of rAPSE expressed in *P. pastoris*.

Table S4. Purification of rAGAL2 expressed in *P. pastoris*.

Table S5. Purification of rAGAL3 expressed in *P. pastoris*.

Table S6. Actions of rAGALs on oligosaccharides.

Table S7. Actions of rAGALs on α-Gal residues in cell walls.

Fig. S1. Schematic diagram of T-DNA insertion sites in *apse*, *agal1*, *agal2*, and *agal3* mutants.

Fig. S2. β-l-Arabinopyranosidase activity in *APSE*- and *AGAL3*-OX plant lines.

Fig. S3. The effect of pH on the activity of rAPSE, rAGAL2, and rAGAL3.

Fig. S4. The expression levels of *AGAL1*, *AGAL2*, *AGAL3*, and *APSE*.

Fig. S5. Action of rAGAL2 and rAGAL3 on mucilage.

Fig. S6. The expression patterns of *AGAL2* and *AGAL3*.

## Supplementary Material

supplementary_tables_S1-S7_figures_S1-S6

## Abbreviations:

AG: arabinogalactan
AGAL1: α-galactosidase 1 from Arabidopsis
AGAL2: α-galactosidase 2 from Arabidopsis
AGAL3: α-galactosidase 3 from Arabidopsis
*AGAL3*-OX: *AGAL3*-overexpressing
AGP: arabinogalactan-protein
l-Ara: l-arabinose
l-Ara*f*: l-arabinofuranose
α-l-Ara*f*: α-l-arabinofuranosyl
β-l-Ara*f*: β-l-arabinofuranosyl
APSE: β-l-ARAPASE from Arabidopsis
*APSE*-OX: *APSE*-overexpressing
l-Ara*p*: l-arabinopyranose
α-l-Ara*p*: α-l-arabinopyranosyl
β-l-Ara*p*: β-l-arabinopyranosyl
GlcA: glucuronic acid
GUS: β-glucuronidase
HPAEC-PAD: high-performance anion-exchange chromatography with pulsed amperometric detection
4-Me-GlcA: 4-*O*-methyl-GlcA
OsAPSE: β-l-arabinopyranosidase from rice
PNP-β-l-Ara*p*: *p*-nitrophenyl-β-l-arabinopyranoside
PNP-α-Gal: *p*-nitrophenyl-α-galactoside
RT: reverse-transcriptase
rAGAL2: recombinant AGAL2
rAGAL3: recombinant AGAL3
rAPSE: recombinant APSE
SaArap27A: β-l-arabinopyranosidase from *Streptomyces avermitilis*.
