# Supplementary material for: Heterologous expression and characterization of an Arabidopsis β-l-arabinopyranosidase and α-d-galactosidases acting on β-l-arabinopyranosyl residues
Source: J Exp Bot. 2017 Aug 8;68(16):4651–61. doi: 10.1093/jxb/erx279 (PMC5853685; doi:10.1093/jxb/erx279)
Supplement: supplementary_tables_S1-S7_figures_S1-S6 [file erx279_suppl_supplementary_tables_s1-s7_figures_s1-s6.pdf]

A GH 27 β-L-arabinopyranosidase and α-D-galactosidases acting on β-L-arabinopyranosyl residues in Arabidopsis

Chiemi Imaizumi, Harumi Tomatsu, Kiminari Kitazawa, Yoshihisa Yoshimi, Seiji Shibano, Kaoru Kikuchi, Masatoshi Yamaguchi, Satoshi Kaneko, Yoichi Tsumuraya, and Toshihisa Kotake

Supplementary Table S1. Sequences of primers used in the present study

Name of primer	Sequence
Primers for genotype determination	
APSE-WT-F1	5’-CTCTCTTACTCTCAGGTTCTCAAC-3’
APSE-WT-R1	5’-ATGTTGTTGCCTCGACCTTGCTTC-3’
AGAL1-WT-F2	5’-TTGCTGGGCTGAAATTTCTCGTGAC-3’
AGAL1-WT-R2	5’-AATGACATGGACGTACCCATTCTGC-3’
AGAL2-WT-F2	5’-CAAGAAATTGACTTGTAAGTGCAGGGG-3’
AGAL2-WT-R2	5’-CTCGGGTCTCGATACAACTTTCTCG-3’
AGAL3-WT-F1	5’-TCAGTCTTGTTCCTCGTCGTAGGC-3’
AGAL3-WT-R1	5’-GAGCTAATTCCTTGAACCTACCCC-3’
LBb1	5’-GCGTGGACCGCTTGCTGCAACT-3’
Primers for heterologous expression in *P. pastoris*	
APSE-F	5’-GCGAATTCAAGGTCGAGGCAACAACATG-3’
APSE-R	5’-GCTGGTACCTCAGGCGTTGCTACAATGAAG-3’
AGAL2-F	5’-GCGAATTCGTTTCAGAGTCGAATGTTGATG-3’
AGAL2-R	5’-CGAGAATTCTTATGCCTTGCGTCTTGTAAG-3’
AGAL3-F	5’-GCGAATTCAGGGAGAGTCAAAGCTCC-3’
AGAL3-R	5’-GCGGAATTCTATACATCAGAGTGTGATAC-3’
Primers for overexpression in Arabidopsis	
APSE-OX-F	5’-CGGGATCCTCTTACTCTCAGGTTCTCAACAAC-3’
APSE-OX-R	5’-CATGAGCTCGACTTTCGATTACACAAGAG-3’
AGAL3-OX-F	5’-CGGGATCCGAAGACACATAGAGGAAGAAG-3’
AGAL3-OX-R	5’-CATGAGCTCTGGCAATTTGTTTAGAGAGTGAC-3’

The restriction sites used for subclonoing are underlined.

Supplementary Table S2. Accession numbers for amino acid sequences.

Abbreviation	Enzyme activity	Organism	Accession numbe
**Prokaryotes**			
AdImdA	Isomaltodextranase	*Arthrobacter dextranolyticus* T6	BAD34648.1
CcGal27A	α-galactosidase	*Clostridium cellulolyticum* H10	ACL75593.1
CjAga27A	α-galactosidase	*Clostridium josui* FERM P-9684	BAB83765.1
CmAga27A	α-galactosidase	*Cellvibrio mixtus* ATCC 12120	AAS19696.1
CpArap27	β-L-arabinopyranosidase	*Chitinophaga pinensis* DSM 2588	ACU63161.1
GsAbp	β-L-arabinopyranosidase	*Geobacillus stearothermophilus* T-6 NCIMB 402	4NX0_A
SaArap27A	β-L-arabinopyranosidase	*Streptomyces avermitilis* MA-4680	BAC69897.1
SeAgaB2	α-galactosidase	*Saccharopolyspora erythraea* NRRL 2338	AAC99325.1
**Eukaryote**			
AfAglB	α-galactosidase	*Aspergillus fumigatus* IMI 385708	ACO72591.1
An7152.2	α-galactosidase	*Aspergillus nidulans* FGSC A4	ABF50881.1
AnAglA	α-N-acetylgalactosaminidase/α-galactosidase	*A. niger* CBS 513.88	CAK44933.1
AnAglB	α-galactosidase	*A. niger* CBS 513.88	CAK44445.1
ArGGT-1	galactan:galactan galactosyltransferase	*Ajuga reptans*	AAR02007.1
AspNagA	α-N-acetylgalactosaminidase	*Acremonium* sp. No.413	BAB08149.1
BspAgalB	α-galactosidase	*Bispora* sp. MEY-1	ADN30030.1
CaAGAL	α-galactosidase	*Coffea arabica*	AAA33022.1
CcCs	β-L-arabinopyranosidase	Citrus clementina	XP_006438231.1
CcGal1	α-galactosidase	*Coffea canephora* 97	CAI47560.1
CsAGAL	α-galactosidase	*Cucumis sativus*	ABC88435.1
CsAPSE	β-L-arabinopyranosidase	*Camelina sativa*	XP_010502554.1
CtAga27A	α-galactosidase	*Cyamopsis tetragonoloba*	CAA32772.1
DhAGAL	α-galactosidase	*Debaryomyces hansenii* CBS767	CAG89932.1
FoAp1	β-L-arabinopyranosidase/α-galactosidase	*Fusarium oxysporum* 12S	BAH10649.1
FoAp2	β-L-arabinopyranosidase/α-galactosidase	*F. oxysporum 12S*	BAH10648.1
GmAGAL	α-galactosidase	*Glycine max*	AAA73963.1
GmAPSE	β-L-arabinopyranosidase	*G. max*	XP_003538597.1
GrAPSE	β-L-arabinopyranosidase	*Gossypium raimondii*	XP_012479301.1
GsAbp	β-L-arabinopyranosidase	*Geobacillus stearothermophilus*	4NX0_A
HaGla	α-galactosidase	*Helianthus annuus*	BAC66445.1
HsGla	α-galactosidase	*Homo sapiens*	AAH02689.1
HsNagA	α-N-acetylgalactosaminidase/α-galactosidase	*H. sapiens*	AAA36351.1
MmGla	α-galactosidase	*Mus musculus*	AAA74453.1
MmNagA	α-N-acetylgalactosaminidase	*M. musculus*	AAC28851.1
MtAga1	α-galactosidase	*Myceliophthora thermophila* C1	AFJ59926.1
NfGal27A	α-galactosidase	*Neosartorya fischeri* P1/CGMCC 3.15369	AGV79321.1
NfGal27B	α-galactosidase	*N. fischeri* P1/CGMCC 3.15369	AJA29661.1
OsαGal I	α-galactosidase	*Oryza sativa* Japonica Group	BAB12570.1
OsαGal II	α-galactosidase	*O. sativa* Japonica Group	BAF21463.1
OsαGal III	α-galactosidase	*O. sativa* Japonica Group	BAC79549.1
OsAPSE	β-L-arabinopyranosidase	*O. sativa* Japonica Group	XP_015621814.1
PcAgalA	α-galactosidase	*Phanerochaete chrysosporium* ME446	AAG24510.1
PsAgl1	α-galactosidase	*Penicillium simplicissimum* VTT-D-78090	CAA08915.1
PvAGAL	α-galactosidase	*Phaseolus vulgaris*	AAA73964.1
PsGal1	α-galactosidase	*Pisum sativum*	CAF34023.1
PeAPSE	β-L-arabinopyranosidase	*Populus euphratica*	XP_011016174.1
ReGal1	α-galactosidase	*Rasamsonia emersonii*	CAF34023.1
ScMEL	α-galactosidase	*Saccharomyces carlsbergensis* NCYC396	AAA34769.1
ScMel1	α-galactosidase	*Saccharomyces cerevisiae*	CAA26888.1
SlGal	α-galactosidase	*Solanum lycopersicum*	AAF04591.1
SmMELj	α-galactosidase	*Saccharomyces mikatae* IFO 1816	CAA64760.1
SpMELp	α-galactosidase	*Saccharomyces paradoxus* 61-248	CAA64759.1
SpMel1	α-galactosidase	*Schizosaccharomyces pombe*	CAB60017.1
TpaGal	α-galactosidase	*Talaromyces purpureogenus* 618	Q13295
TdMELt	α-galactosidase	*Torulaspora delbrueckii*	BAA86883.1
TrAgl1	α-galactosidase	*Trichoderma reesei* RUTC-30	CAA93244.1
TrAgl3	α-galactosidase	*T. reesei* RUTC-30	CAA93244.1
UvAGAL1	α-galactosidase	*Umbelopsis vinacea*	AAB35252.2
UvAGAL2	α-galactosidase	*U. vinacea*	BAA33931.1
VvAPSE	β-L-arabinopyranosidase	*Vitis vinifera*	XP_002282325.1
ZmMELr	α-galactosidase	*Zygotorulaspora mrakii* IFO 1835	BAA99555.1

Supplementary Table S3. Purification of rAPSE expressed in *P. pastoris*

	Total protein	Total activity[a]	Specific activity	Purification	Yield
	mg	*units*	*units mg protein^{-1}*	*-fold*	*%*
Culture medium	48	2.9	0.06	1.0	100
CM-Sepharose FF	1.2	0.7	0.58	9.7	24

[a] Enzyme activity was measured using 1 mM PNP-β-L-Ara*p* as the substrate.

Supplementary Table S4. Purification of rAGAL2 expressed in *P. pastoris*

	Total protein	Total activity[a]	Specific activity	Purification	Yield
	mg	*units*	*units mg protein^{-1}*	*-fold*	*%*
Culture medium	28.6	405	14.2	1.0	100
CM-Sepharose FF	10.0	1059[b]	106	7.5	261
Sephacryl S-200	0.24	23	95	6.7	5.7

[a] Enzyme activity was measured using 1 mM PNP-α-Gal as the substrate.

[b] The drastic increase in total activity may result from the removal of inhibitory substance(s) included in the culture medium.

Supplementary Table S5. Purification of rAGAL3 expressed in *P. pastoris*

	Total protein	Total activity[a]	Specific activity	Purification	Yield
	mg	*units*	*units mg protein^{-1}*	*-fold*	*%*
Culture medium	46	64	1.4	1.0	100
DEAE-Sepharose FF	14	58	4.1	2.9	77
Sephacryl S-200	4.4	28	6.4	4.6	48

[a] Enzyme activity was measured using 1 mM PNP-α-Gal as the substrate.

Supplementary Table S6. Actions of rAGALs on oligosaccharides

Substrate[a]	rAGAL2[b]	rAGAL3[b]
PNP-α-Gal	100.0	100.0
Raffinose	1.9	0.4
Stachyose	1.7	0.7

[a]The enzyme was incubated with 1 mM PNP-α-Gal or 5 mg/mL oligosaccharide at 37°C. Raffinose {α-Gal-(1→6)-α-Glc-(1→2)-β-fructofuranoside} and stachyose {α-Gal-(1→6)-α-Gal-(1→6)-α-Glc-(1→2)-β-fructofuranoside} were from Wako (Tokyo, Japan).

[b]Activity is expressed in % of that toward PNP-α-Gal.

Supplementary Table S7. Actions of rAGALs on α-Gal residues in cell walls

Substrate	Released Gal (nmol/mg AIR)[a]	
	rAGAL2[b]	rAGAL3[b]
Aerial parts of seedlings[b]	0.04	0.04
Roots[b]	0.04	0.01
Rosette leaves[b]	0.01	<0.01
Inflorescence[b]	0.02	0.27
Siliques[b]	0.02	<0.01
Seeds with mucilage[c]	1.46	1.85

[a]The cell wall fraction or mucilage was incubated with recombinant enzyme at pH 5.0 at 37°C for 24 h, and the released Gal was measured.

[b]The cell wall fractions were prepared from aerial parts and roots of seedlings grown on MS-agar medium for 2 weeks, and the rosette leaves, stems and siliques of matured plants grown on rockwool fiber for 5 weeks as described (Fry, 2001; Harholt *et al.*, 2006).

[c]Arabidopsis seeds were imbibed in water for 3 h, heated at 100°C for 5 min to inactivate endogenous enzymes, and incubated with recombinant enzyme (details are described in the legend to Supplementary Fig. S4).

Fry S. 2001. Chemical and Metabolic Analysis. Caldwell, NJ: The Blackburn Press; The Growing Plant Cell Wall.

Harholt J, Jensen JK, Sørensen SO, Orfila C, Pauly M, Scheller HV. 2006. ARABINAN DEFICIENT 1 is a putative arabinosyltransferase involved in biosynthesis of pectic arabinan in Arabidopsis. Plant Physiology **140,** 49-58.

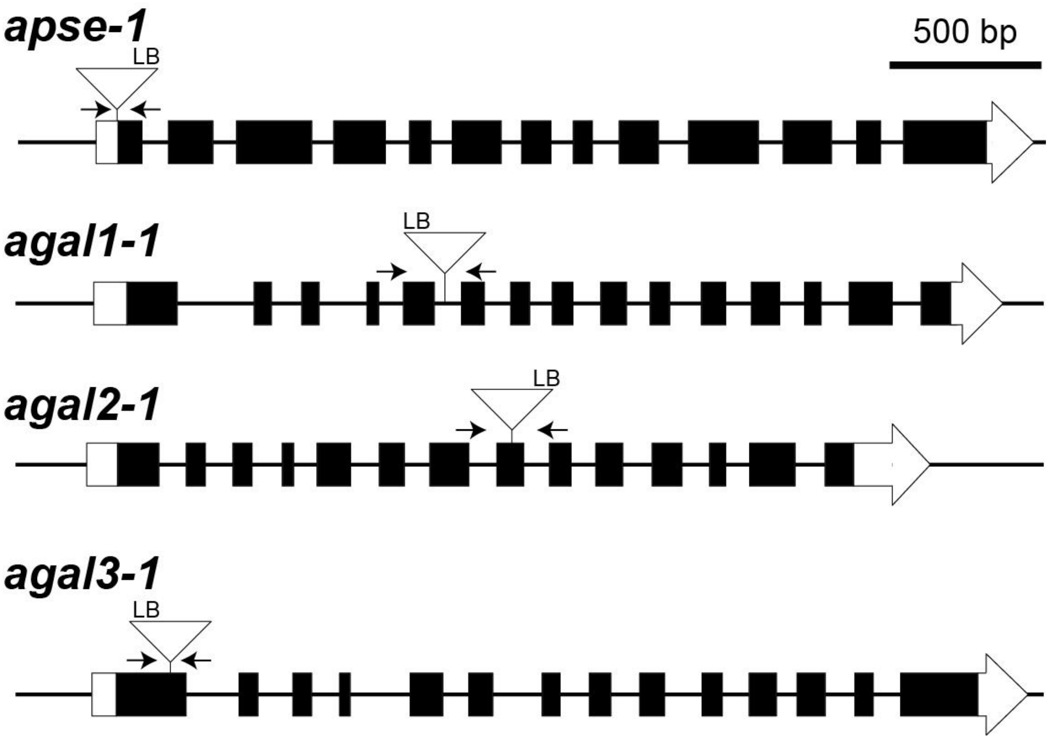

Supplementary Figure S1. Schematic diagram of T-DNA insertion sites in *apse*, *agal1*, *agal2*, and *agal3* mutants. Arrows indicate primers used for the determination of genotype that are listed in Supplementary Table S1.

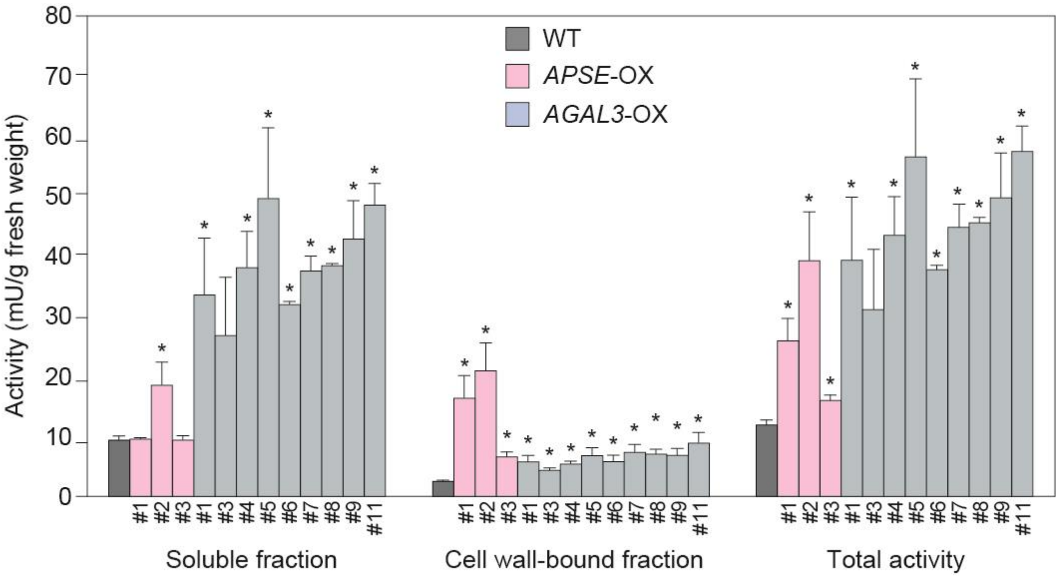

Supplementary Figure S2. β-L-Arabinopyranosidase activity in *APSE*– and *AGAL3*–OX plant lines. Data are mean values with standard error (n = 3 biological replicates). The asterisk indicates significant difference from WT plant (Student's t-test, $p < 0.05$).

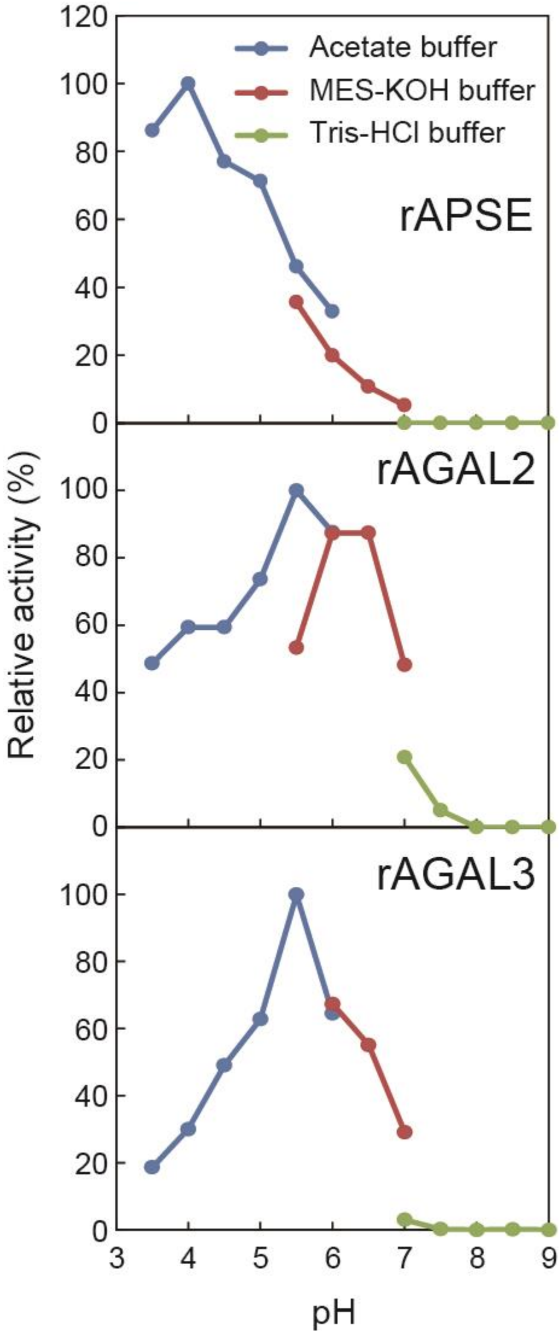

Supplementary Figure S3. The effect of pH on the activity of rAPSE, rAGAL2, and rAGAL3. Activity-pH curves result from experiments with reaction mixtures including 50 mM buffer and 1 mM PNP-substrate.

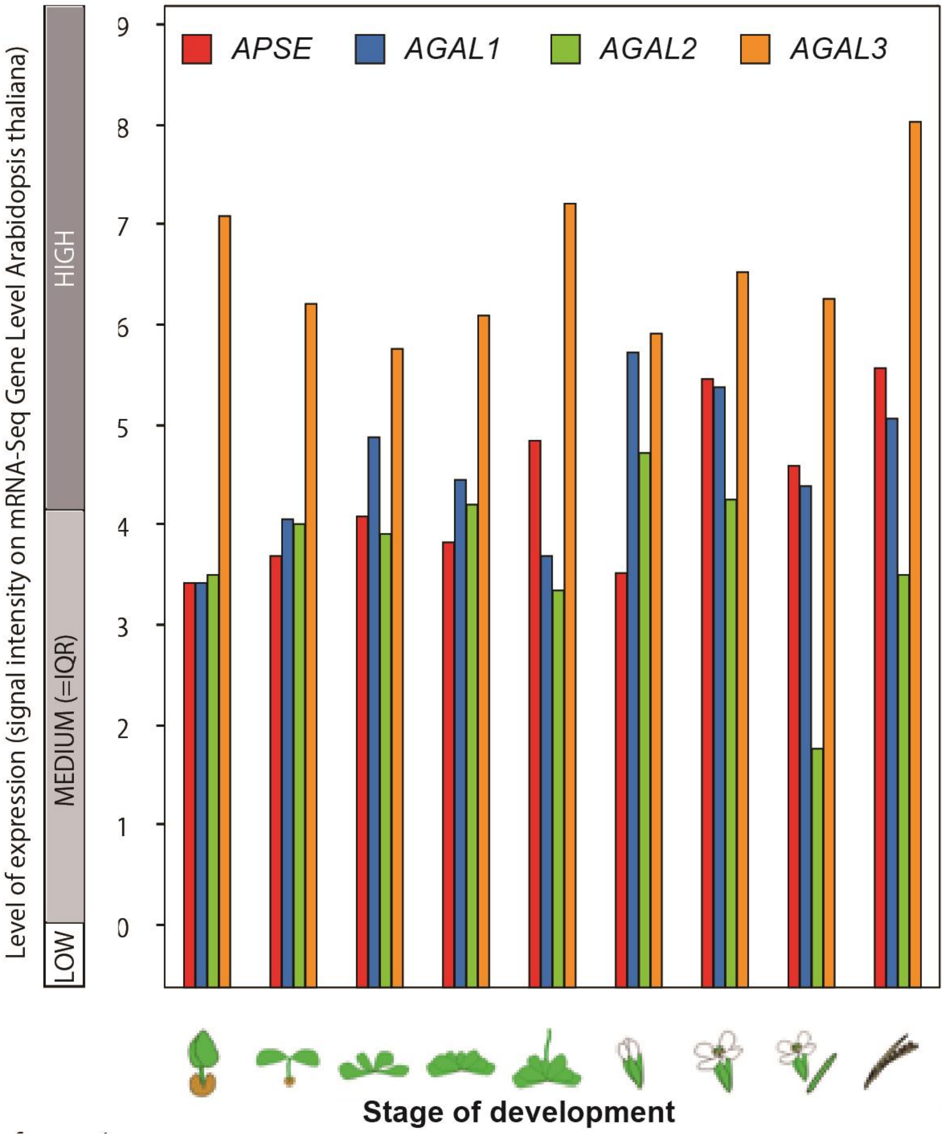

Supplementary Figure S4. Expression levels of *AGAL1*, *AGAL2*, *AGAL3*, and *APSE*. The data were acquired from Genevestigator. Based on the data, the expression level of *AGAL3* is higher than those of *AGAL1*, *AGAL2*, and *APSE*.

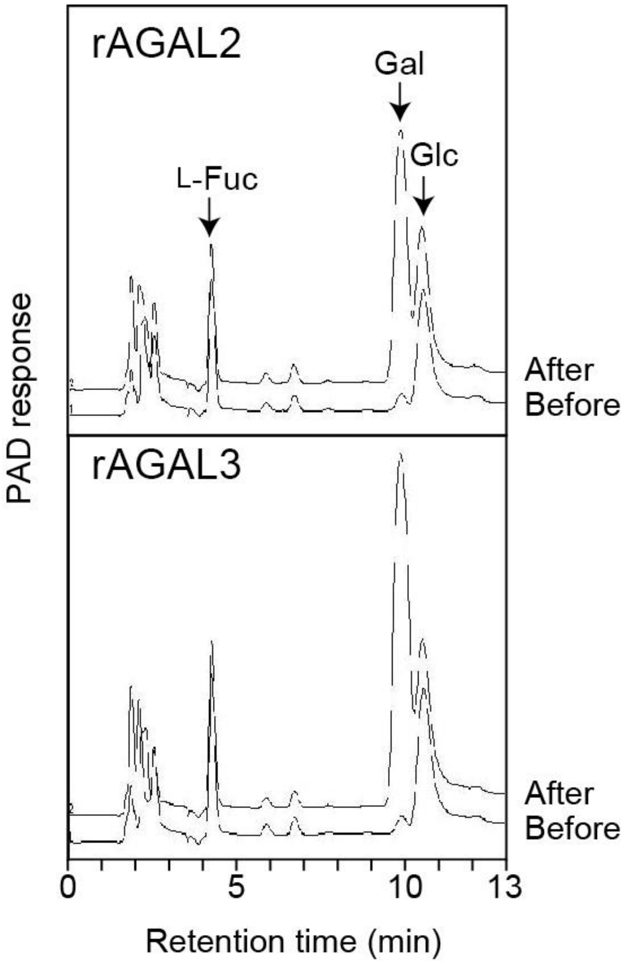

Supplementary Figure S5. Action of rAGAL2 and rAGAL3 on mucilage. Arabidopsis seeds (app. 40 mg) were sterilized with 50% (w/w) sodium hypochlorite. The mucilage was released by shaking with vortex at maximum speed for 3 h at 25°C and treated at 100°C for 5 min to inactivate endogenous enzymes. The reaction was performed with a mixture (1 mL) containing 50 mM sodium acetate buffer (pH 5.0), 10 munits of rAGAL2 or rAGAL3, and the seeds with mucilage at 37°C for 24 h. The reaction was terminated by heating at 100°C for 5 min, and then L-Fuc was added to the mixture as the calibration standard. Gal released from mucilage was measured by HPAEC-PAD. The quantified values are shown in Supplementary Table S7.

AGAL2

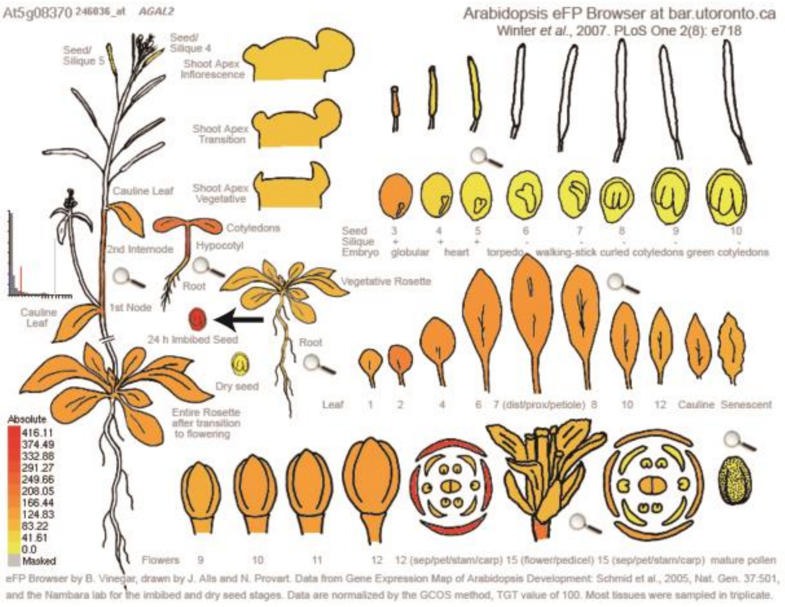

AGAL3

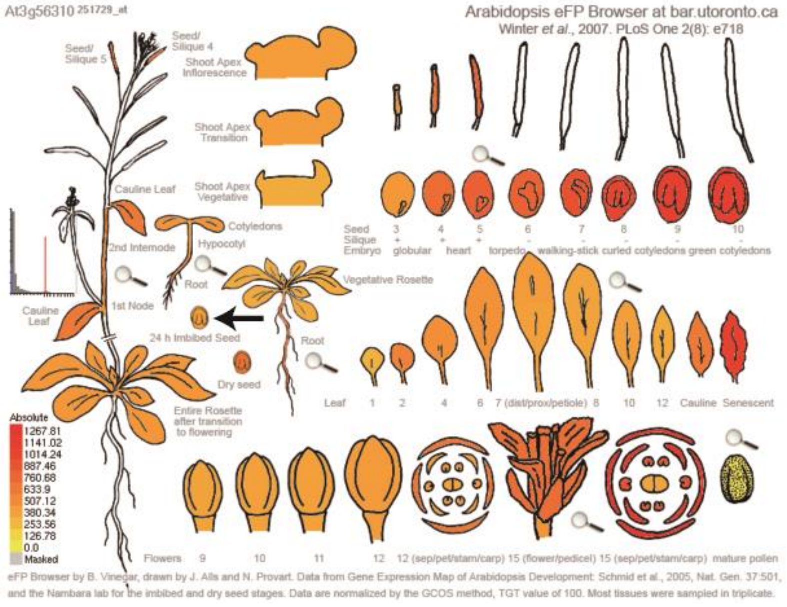

Supplementary Figure S6. The expression patterns of *AGAL2* and *AGAL3*. The data were acquired from the Arabidopsis eFP browser. The relative expression level of *AGAL2* is high and that of *AGAL3* is moderate in imbibed seeds, which are indicated by arrows.
